# Wearing complete dental prostheses - Effects on perioral morphology

**DOI:** 10.4317/medoral.21102

**Published:** 2016-03-31

**Authors:** Gregor F. Raschke, Philipp Eberl, Geoffrey A. Thompson, Arndt Güntsch, Andre Peisker, Stefan Schultze-Mosgau, Marta Gomez-Dammeier, Gabriel Djedovic, Ulrich M. Rieger, Florian Beuer

**Affiliations:** 1 MD, DDS, PhD. MD, DDS. MD, DDS, PhD. DMD. Department of Cranio-Maxillofacial & Plastic Surgery, Friedrich Schiller University Jena, Germany; 2 DMD. DMD, PhD. Department of Prosthodontics, Geriatric Dentistry, and Craniomandibular Disorders, Charité Berlin, Germany; 3 DDS, MS, PhD. DMD, PhD. Marquette University School of Dentistry, Milwaukee, WI, USA; 4 MD. MD, PhD. Department of Plastic & Aesthetic, Reconstructive & Hand Surgery, St. Markus Hospital, Johann Wolfgang von Goethe University, Frankfurt/Main, Germany

## Abstract

**Background:**

To adequately perform rehabilitation of edentulous patients by a complete removable dental prosthesis (CRDP) is from basic interest to dentists to understand the morphologic changes caused by re-establishment of a physiologic jaw relationship. Anthropometric analyses of standardized frontal view and profile photographs may help elucidate such changes.

**Material and Methods:**

Photographs of 31 edentulous patients were compared in relaxed lip closure and after insertion of a CRDP in stable occlusion. 2232 anthropometric distances were raised. Eighteen anthropometric indices reflecting the perioral morphology and its integration in the vertical facial harmony were investigated.

**Results:**

The intercanthal – mouth width index (*p*<.001), medial - lateral cutaneous upper lip height index (*p*=.007), lower vermilion contour index (*p*=.022), vermilion - total upper lip height index (*p*=.018), cutaneous - total upper lip height index (*p*=.023), upper lip - nose height index (*p*=.001), nose - upper face height index (*p*=.002), chin - mandible height index (*p*=.013), upper lip - mandible height index (*p*=.045), nose - lower face height index (*p*=.018), and nose - face height index (*p*=.029) showed significant pre- to post-treatment changes.

**Conclusions:**

The investigated anthropometric indices presented reproducible results related to an increase in occlusal vertical dimension. Their application may be helpful in assessment, planning, and explanation of morphologic effects of CRDPs on the perioral and overall facial morphology, which may helps to improve the aesthetic outcome.

**Key words:**Dentures, removable dentures, anthropometry, perioral morphology.

## Introduction

Worldwide, conventional complete removable dental prostheses are the most established form of treatment of edentulous jaws (1). Most patients treated with CRDPs are satisfied with the increase in quality of life achieved. The perception of gain of quality of life maybe considered as the most established indicator for a successful therapy (2-5).

Today, patients exhibit an increased awareness not only of the functional but also of the aesthetic quality of their artificial dentition. To ensure an adequate aesthetic appearance of CRDPs, there are established clinical methods to perform e.g. an aesthetic anterior tooth arrangement or selection of tooth colour (6,7). Beside these techniques to improve the oral appearance of CRDPs it is beyond dispute, that CRDPs have also an immense influence on patients´ overall facial appearance.

Despite this knowlegde, there is no established objective and reproducible procedure to evaluate the influence of CRDPs on the perioral and facial morphology.

Anthropometric indices described by Leslie G. Farkas provide objective and relative information about relationships between at least two anthropometric distances (8-10). They have proven useful to objectively quantify changes in the facial morphology by reconstructive (11), traumatologic (12) and orthognathic (13) surgery. Furthermore, they are used in the description of perioral aging as well as of planning facial changes e.g. in orthodontics (14-16).

In the present study the effect of CRDPs on anatomic landmarks and perioral relationships on standardized frontal view and profile photographs in edentulous patients was investigated. Results of photo-assisted anthropometric measurements of edentulous patients without CRDP in relaxed lip closure and with their CRDP in situ were compared.

## Material and Methods

Before the study was initiated, the local ethic committee gave its approval. All patients willing to participate signed consent before being enrolled.

Patients exhibiting specific or general diagnoses potentially influencing the perioral architecture, like e.g. earlier facial operations, were excluded. All patients were edentulous and received a conventional CRDP at the Dental Clinic in a standardized manner (17). At the time the photographs were taken all patients exhibited a stable occlusion in all quadrants, when CRDP was inserted.

- Objective Rating Scheme

Coloured frontal view and profile photographs with open eyes were taken of the patient’s by a professional photographer (Nikon D 80 camera; objective: Nikon AF Micro Nikkor 105 mm 1: 2.8 D; aperture: f13; Nikon Corp, Tokyo, Japan). Photographs were made after the CRDP were inserted, stable, and with lips in a relaxed position. Only photographs showing patients´ faces clearly at rest and in which the interpupillary axis was at the same level as the camera lens were selected to avoid photographic distortion (18). Photographic analysis was performed using Adobe Photoshop CS2 (Adobe Inc, San Jose, CA) software measurement tool.

**Figure 1:**
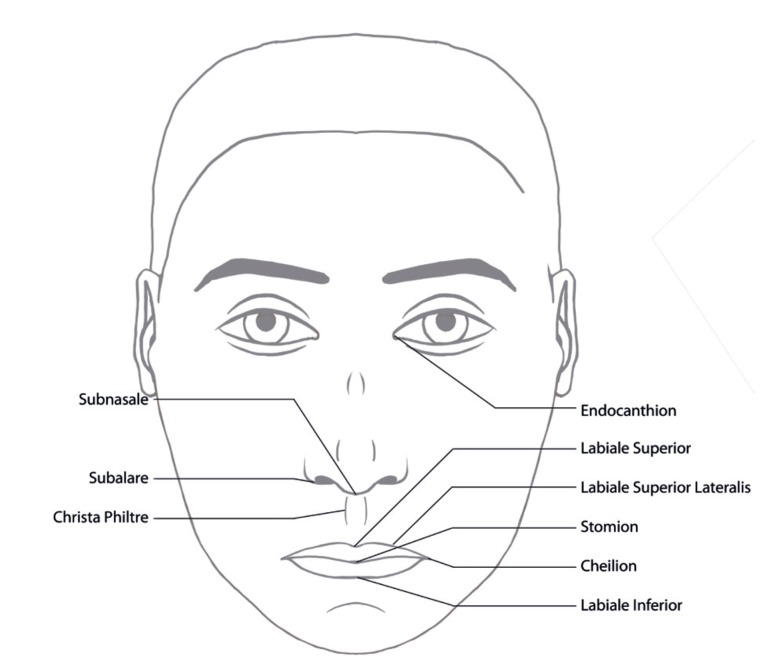
Schematic frontal-view image with description of the used anthropometric points.

On the basis of predefined landmarks ( Table 1) and data ( Table 2), the following anthropometric indices based on the work of Farkas and Munro (9,10) were investigated (see also Fig. 1):

**Table 1:**
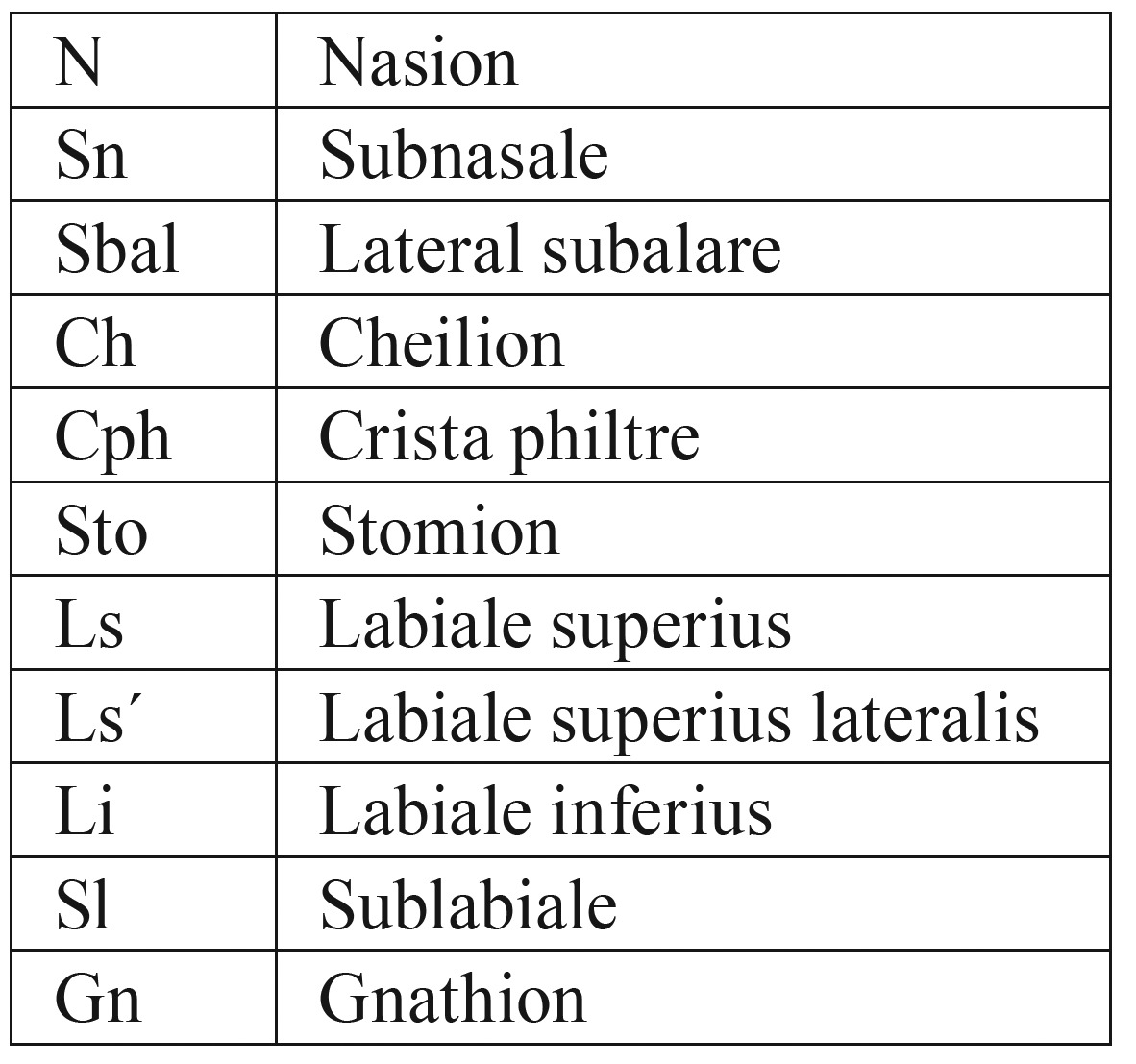
Used anthropometric landmarks based on the investigations by Farkas and Munro.

**Table 2:**
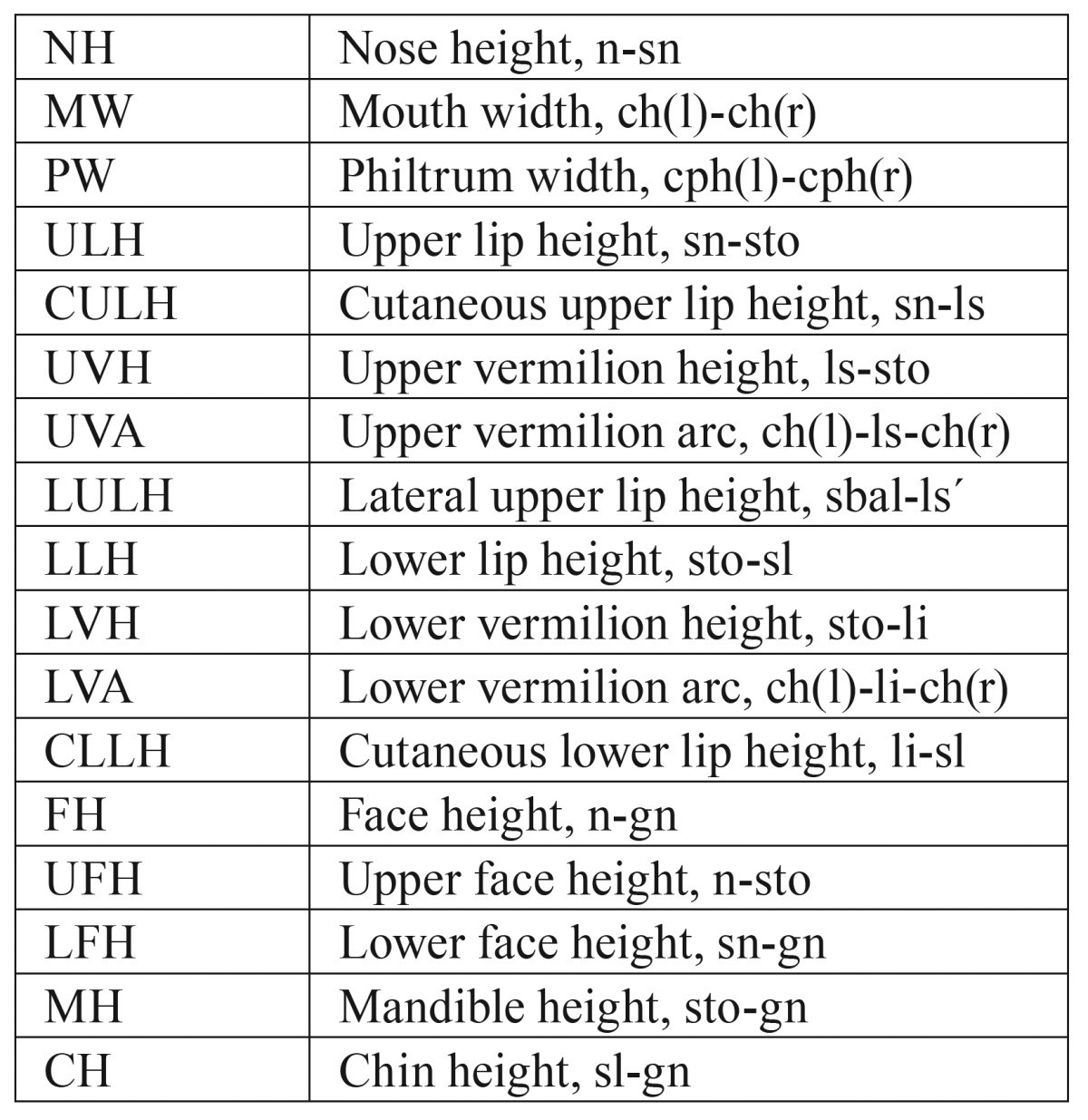
Used anthropometric distances based on the investigations by Farkas and Munro.

(1) Intercanthal-mouth width index, representing the intercanthal width (IW, en-en), as a percentage of the mouth width (MW, ch-ch) (2); Upper lip height mouth width index, representing the upper lip height, the vertical distance between the subnasale and the stomion (ULH, sn-sto) as a percentage of the mouth width (MW, ch-ch) (3); Medial-lateral cutaneous upper lip height index representing the cutaneous upper lip height, the vertical distance between the labiale superius and the subnasale (CULH, sn-ls), as a percentage of the lateral upper lip height, the vertical distance between the subalare and the lateral labiale superius beyond the subalare (LULH, sbal-ls´) (4); Upper vermilion contour index, the mouth width (MW) as a percentage of the upper vermilion arc (UVA, ch-ls-ch) (5); Lower vermilion contour index, the mouth width (MW) as a percentage of the lower vermilion arc (LVA, ch-li-ch); and (6) Vermilion arc index, the lower vermilion arc (LVA) as a percentage of the upper vermilion arc (UVA).

**Figure 2:**
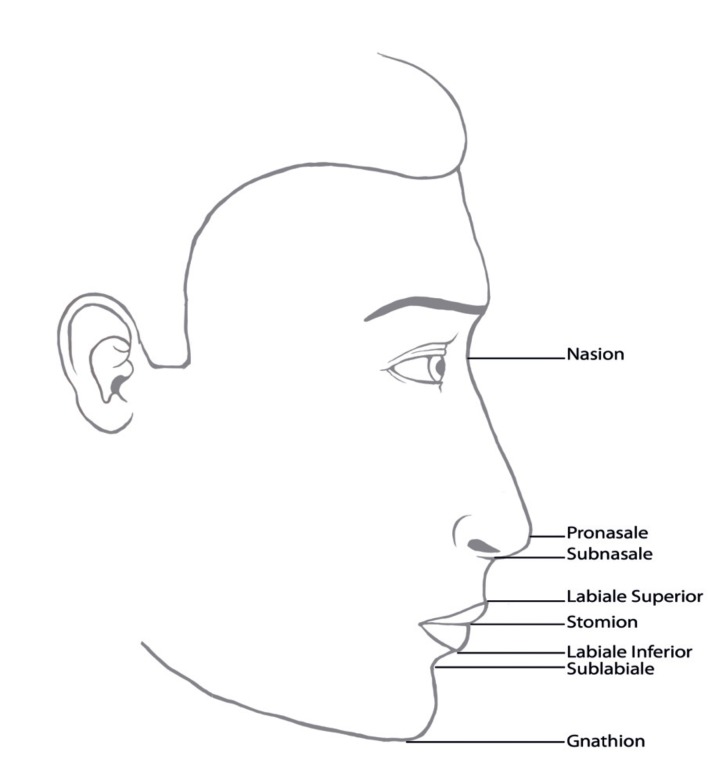
Schematic profile-view image with description of the used anthropometric points.

In the profile photographs the following data were recorded (see also Fig. 2): (1) Vermilion total lower lip height index, the lower vermilion height, the vertical distance between stomion and labiale inferius (LVH, sto-li) as a percentage of the lower lip height (LLH, sto-sl) (2); Vermilion total upper lip height index represented by the upper vermilion height, the vertical distance between labiale superius and stomion (UVH, ls-sto), as a percentage of the upper lip height (ULH, sn-sto) (3); Cutaneous total lower lip height index represented by the cutaneous lower lip height, the vertical distance between the labiale inferius and the sublabiale (CLLH, li-sl), as a percentage of the lower lip height, the vertical distance between the stomion and the sublabiale (LLH, sto-sl) (4); Cutaneous total upper lip height index, the vertical distance between cutaneous upper lip height (CULH, sn-ls) as a percentage of the upper lip height, the vertical distance between subnasale and stomion (ULH, sn-sto) (5); Vermilion height index, represented by the upper vermilion height (UVH, ls-sto), as a percentage of the lower vermilion height (LVH, sto-li) (6); Upper lip- nose height index, the upper lip height (ULH, sn-sto), as a percentage of the nose height (NH, n-sn) (7); Nose- upper face height index, the nose height (NH, n-sn), as a percentage of the upper face height (UFH, n-sto) (8); Chin- mandible height index, the chin height (CH, sl-gn), as percentage of the mandible height (MH, sto-gn) (9); Upper face- face height index, the upper face height (UFH, n-sto), as a percentage of the face height (FH, n-gn) (10); Upper lip- mandible height index, representing the upper lip height (ULH, sn-sto), as a percentage of the mandible height (MH, sto-gn) (11); Nose- lower face height index, the nose height (NH, n-sn), as a percentage of the lower face height (LFH, sn-gn) and (12); Nose- face height index, the nose height (NH, n-sn), as a percentage of the face height (FH, n-gn).

- Statistical Analysis

All calculations were done using SPSS V 19.0 for Windows (SPSS, Inc, Chicago, IL). Data are presented as mean and standard deviation. To compare results of the performed anthropometric measurements without CRDP in relaxed lip closure and with inserted CRDP the students t-test was applied. A *p*-value of <0.05 was taken as significant.

## Results

31 patients, 19 (61.3%) men and 12 (38.7%) women, were included in this study. Mean age was 61.53±7.15 at the time photographs were taken.

Overall 2232 anthropometric distances were measured to determine 1116 anthropometric indices. Results of the photo-assisted anthropometric measurements with and without CRDP are given in t Table 3.

**Table 3:**
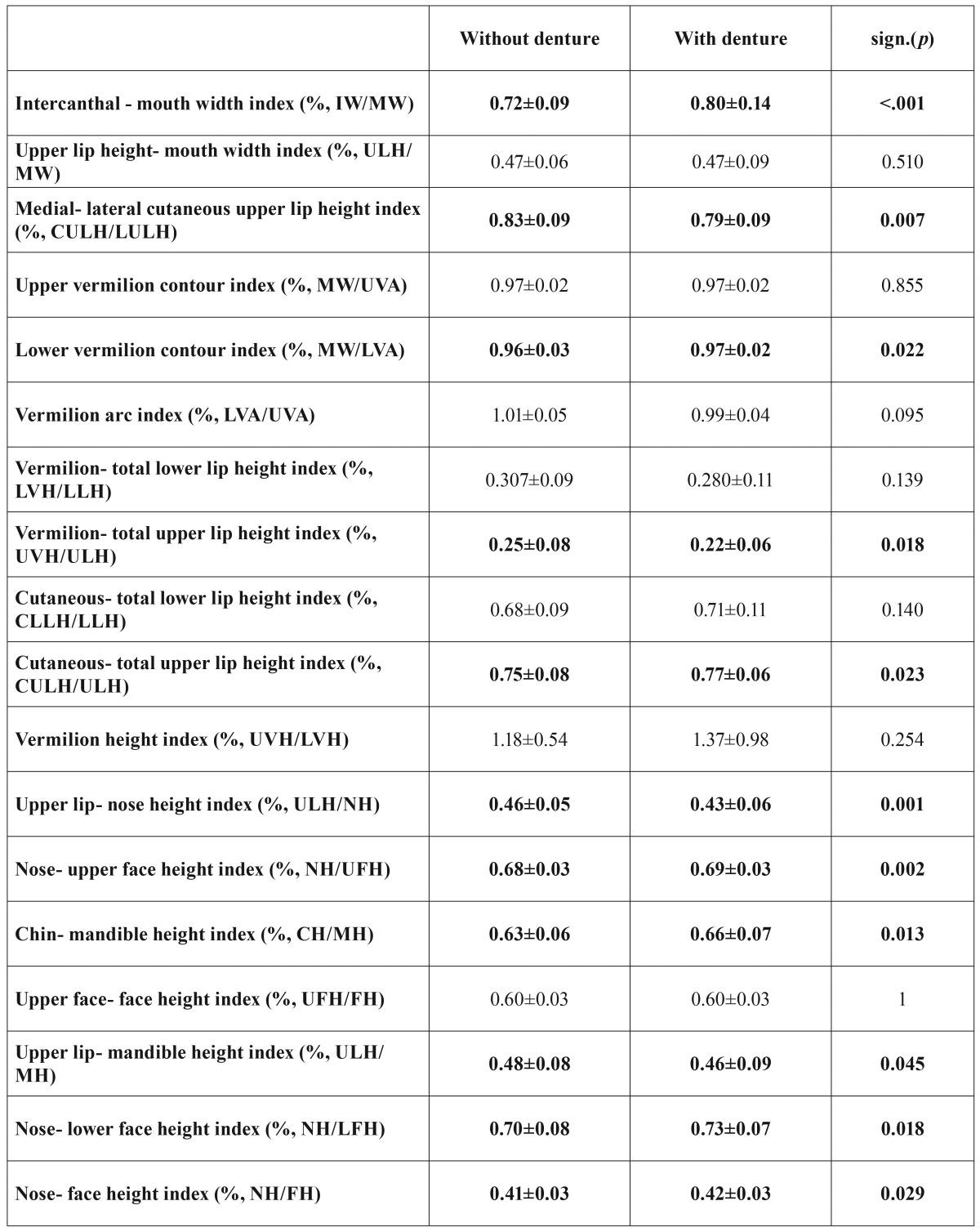
Comparison of the results of anthropometric measurements with and without CRDP.

The intercanthal-mouth width index (*p*<.001), medial-lateral cutaneous upper lip height index (*p*=.007), lower vermilion contour index (*p*=.022), vermilion- total upper lip height index (*p*=.018), cutaneous-total upper lip height index (*p*=.023), upper lip-nose height index (*p*=.001), nose-upper face height index (*p*=.002), chin-mandible height index (*p*=.013), upper lip-mandible height index (*p*=.045), nose-lower face height index (*p*=.018), and nose-face height index (*p*=.029) showed significant differences, when a CRDP was inserted.

- Discussion of the Method

Edentulous patients present various changes in their facial morphology related to anatomic changes due to tooth loss. These changes seem to affect the lower face especially. Due to loss in the alveolar ridge height patients show a tendency towards a Class III relationship of the jaws, reduction of the vertical relationships between lower and upper face, and an increased prominence of the chin (6,19-22). Also the upper and lower vermilion present a collapsed aspect in edentulous patients, as the former inner dental support of the lips is absent (6). Due to this morphologic changes the physiologic rest position seems to undergo a subtle process of neuromuscular adaptation which leads to a decreased vertical relation of the jaws (20).

Knowledge and a complete understanding of facial changes achievable by CRDPs are fundamental to perform a successful functional and aesthetic rehabilitation of edentulous patients (23,24). Given patients´ increased demand on an aesthetic appearance it seems occasionally to be more important to achieve an adequate endorsement by the patient of the perioral soft tissues than to focus on the appearance of the teeth themselves (25-27).

Thus, the correct determination of the vertical relation is of greatest importance (28). It serves to determine the correct relation and redefine the physiological level of occlusion. Despite this key role, there is no established method to reproducibly and objectively determine the vertical relation of the jaws in edentulous patients.(19,28).

In this context, anthropometric measurements may help to approximate the correct vertical relation. A deeper understanding of the morphology of the facial soft tissues, together with established intraoral measurements, may be particularly helpful to younger colleagues to explain the impact of CRDPs on the perioral morphology and to successfully plan CRDPs as well as to discuss with the patient the expected outcome.

Multiple anthropometric and anatomic relations of the perioral region are available. The selected anthropometric indices by Farkas were found useful in earlier studies investigating the effect of orthognathic and reconstructive surgery or aging on the perioral soft tissues (16,29,30), and in the present study were adequate to investigate the complex aesthetic interactions in the aesthetic units of upper and lower lips and their subunits. All anthropometric landmarks were easily and reproducibly identifiable on the standardized photographs.

Intercanthal - mouth width index describes the relationship of the perioral region to the upper central face. It gives a direct reflection of the aspect of the mouth width, which often seems to be decreased in patients with a collapsed vertical relation (9).

The upper lip height - mouth width index investigates the vertical extension of the upper lip to the horizontal extension of the mouth width. The medial - lateral cutaneous upper lip height index investigates the relation of the medial to the lateral vertical extension of the upper lip. Both indices are important in the judgement of the correct configuration of the upper lip as they combine vertical and horizontal relations.

The major feature of both, upper and lower lips, are the vermilions and their relation to each other (31). Considerations of their dimension and composition are highly relevant for planning CRDPs, as they build the aesthetically highly important junction between the internal oral mucosa and the external white skin.

In the frontal view photographs, upper and lower vermilion contour index as well as the vermilion arc index describe relations between upper and lower vermilion and mouth width. In the profile photographs the vermilion height index investigates the vertical relation of the upper and lower vermilion height.

The vermilion and cutaneous total upper and lower lip height indices describe the relation of the vermilion and cutaneous height to the overall height of upper and lower lip. Reflections about these relations are from bigger importance as they are from bigger influence on the perioral aesthetic appearance and they all seem influenceable by CRDPs.

In order to quantify the change in position of the physiologic rest position from its unnatural collapsed position we investigated vertical relations of face height, upper face height, nose height, upper lip height, lower face height, mandible height, and chin height.

Because this study aimed to investigate changes in the facial morphology due to an increase in occlusal vertical dimension by conventional CRDPs men and women were not differentiated between.

- Discussion of the Results

The intercanthal - mouth width index showed a significant decrease, when CRDP was inserted. This result originates from the aspect of a decreased mouth width, when CRDP was inserted. The lacking vertical support of the perioral soft tissues often leads to collapse of the the oral mouth opening surrounding soft tissues and subsequent the aspect of an decreased mouth width in edentulous patients. Insertion of a denture should lead to re-establishment and inner support of the perioral soft tissues and the nasolabial fold, which should be raised to a more original and natural dimension.

The significant increase of the medial – lateral cutaneous upper lip height index gives more detailed information about the distribution of the dimension of re-establishment of soft tissue architecture in the upper lip. Its increase is primarily caused by a decrease of the lateral cutaneous upper lip height. The medial upper lip height is supported by the osseous nasospinale and less affected by the collapse of soft tissues than the lateral portion in edentulous patients. Subsequently, CRDPs´ support of soft tissues is more effective in the lateral aspect of the cutaneous upper lip and leads to its vertical decrease.

Regarding the relation of the white cutaneous upper lip height and the total upper lip height we found a significant decrease of the cutaneous portion of the upper lip. This disproportionate increase in comparison to the total upper lip height and the increased upper vermilion height finds its expression also in the significant increase of the vermilion - total upper lip height index. The re-establishment of the vertical relation of the upper lips leads especially to an increased height of the upper vermilion. This is from special interest when patients are asking for their extraoral aesthetic rehabilitation, in which the upper vermilion is from special interest as its increase leads to a younger and more attractive aspect. This should be considered especially in female patients.

In contrast to the upper lips, the lower lips present an unchanged vertical relation of cutaneous and red portions when CRDP is inserted, as indicate the not significant influenced vermilion - total lower lip height index and cutaneous - total lower lip height index. Also the relation of the vertical relation of the upper to the lower vermilion remains unchanged, as indicates the not significant influenced vermilion height index.

The perioral architecture is not only influenced by vertical rehabilitation, also the overall vertical facial harmony is significantly influenced when a CRDP is inserted.

The significant increase of the upper lip - nose height index and the significant decrease of the nose – upper face height index reflect an increased vertical height of the upper lip towards other midfacial vertical proportions, as the collapsed soft tissues of the upper lip have regained its inner dental support and vertical position more downward.

The significant decrease of the chin - mandible height index, nose - lower face height index, and nose – face height index reflect the vertical increase of the mandible height due to downward rotation within the scope to re-establish the physiological rest position. Overall, this leads to more gain in vertical height of the upper lip than of the lower lip, as indicates the significant increased upper lip – mandible height index.

The presented photo-assisted anthropometric measurements may help to understand the direction and extent of morphological changes in the rehabilitation not only of edentulous jaws but also of the edentulous face.

## Conclusions

The evaluation of the effects of conventional CRDPs on the facial morphology of edentulous patients by using anthropometric data extracted from standardized photographs helps to accurately analyze and quantify treatment related changes. The vertical height of the upper and lower lip and the upper vermilion show a significant increase meanwhile upper lip´s lateral portion decreases in height. Support of the soft tissues surrounding the mouth opening leads to a decreased aspect of the mouth width.

## References

[1] Carlsson GE, Omar R (2010). The future of complete dentures in oral rehabilitation. A critical review. J Oral Rehabil.

[2] Charles Finn J, Cox SE, Earl ML (2003). Social implications of hyperfunctional facial lines. Dermatol Surg.

[3] VAN Lierde K, Browaeys H, Corthals P, Mussche P, VAN Kerkhoven E, DE Bruyn H (2012). Comparison of speech intelligibility, articulation and oromyofunctional behaviour in subjects with single-tooth implants, fixed implant prosthetics or conventional removable prostheses. J Oral Rehabil.

[4] Garrett NR, Kapur KK, Perez P (1996). Effects of improvements of poorly fitting dentures and new dentures on patient satisfaction. J Prosthet Dent.

[5] van Waas MA (1990). Determinants of dissatisfaction with dentures: a multiple regression analysis. J Prosthet Dent.

[6] Nairn RI (1965). Interrelated factors in complete denture construction. J Prosthet Dent.

[7] Mavroskoufis F, Ritchie GM (1980). The face-form as a guide for the selection of maxillary central incisors. J Prosthet Dent.

[8] Edler R, Agarwal P, Wertheim D, Greenhill D (2006). The use of anthropometric proportion indices in the measurement of facial attractiveness. Eur J Orthod.

[9] Farkas LG, Katic MJ, Hreczko TA, Deutsch C, Munro IR (1984). Anthropometric proportions in the upper lip-lower lip-chin area of the lower face in young white adults. Am J Orthod.

[10] Farkas LG, Hreczko TA, Kolar JC, Munro IR (1985). Vertical and horizontal proportions of the face in young adult North American Caucasians: revision of neoclassical canons. Plast Reconstr Surg.

[11] Raschke GF, Rieger UM, Bader RD, Kirschbaum M, Eckardt N, Schultze-Mosgau S (2012). Evaluation of nasal reconstruction procedures results. J Craniomaxillofac Surg.

[12] Raschke G, Rieger U, Bader RD, Schaefer O, Guentsch A, Schultze-Mosgau S (2012). Outcomes analysis of eyelid deformities using photograph-assisted standardized anthropometry in 311 patients after orbital fracture treatment. J Trauma Acute Care Surg.

[13] Raschke GF, Rieger UM, Bader RD, Guentsch A, Schaefer O, Schultze-Mosgau S (2013). Soft tissue outcome after mandibular advancement--an anthropometric evaluation of 171 consecutive patients. Clin Oral Investig.

[14] Gosman SD (1950). Anthropometric method of facial analysis in orthodontics. Am J Orthod.

[15] Liou EJ, Subramanian M, Chen PK (2007). Progressive changes of columella length and nasal growth after nasoalveolar molding in bilateral cleft patients: a 3-year follow-up study. Plast Reconstr Surg.

[16] Raschke GF, Rieger UM, Bader RD, Schaefer O, Guentsch A, Gomez Dammeier M (2014). Perioral aging-an anthropometric appraisal. J Craniomaxillofac Surg.

[17] Divaris K, Ntounis A, Marinis A, Polyzois GL, Polychronopoulou A (2012). Patients' profiles and perceptions of complete dentures in a university dental clinic. Int J Prosthodont.

[18] Flowers RS, Flowers SS (1993). Diagnosing photographic distortion. Decoding true postoperative contour after eyelid surgery. Clin Plast Surg.

[19] Sierpinska T, Golebiewska M, Kuc J, Lapuc M (2009). The influence of the occlusal vertical dimension on masticatory muscle activities and hyoid bone position in complete denture wearers. Adv Med Sci.

[20] Nakai N, Abekura H, Hamada T, Morimoto T (1998). Comparison of the most comfortable mandibular position with the intercuspal position using cephalometric analysis. J Oral Rehabil.

[21] Divaris K, Ntounis A, Marinis A, Polyzois G, Polychronopoulou A (2012). Loss of natural dentition: multi-level effects among a geriatric population. Gerodontology.

[22] Chou JC, Thompson GA, Aggarwal HA, Bosio JA, Irelan JP (2014). Effect of occlusal vertical dimension on lip positions at smile. J Prosthet Dent.

[23] Carruthers JD, Glogau RG, Blitzer A, Facial Aesthetics Consensus Group Faculty (2008). Advances in facial rejuvenation: botulinum toxin type a, hyaluronic acid dermal fillers, and combination therapies-consensus recommendations. Plast Reconstr Surg.

[24] Coleman SR, Grover R (2006). The anatomy of the aging face: volume loss and changes in 3-dimensional topography. Aesthet Surg J.

[25] Hantash RO, AL-Omiri MK, Yunis MA, Dar-Odeh N, Lynch E (2011). Relationship between impacts of complete denture treatment on daily living, satisfaction and personality profiles. J Contemp Dent Pract.

[26] Pivonkova V, Rubesova A, Lindova J, Havlicek J (2011). Sexual dimorphism and personality attributions of male faces. Arch Sex Behav.

[27] Langlois JH, Kalakanis L, Rubenstein AJ, Larson A, Hallam M, Smoot M (2000). Maxims or myths of beauty? A meta-analytic and theoretical review. Psychol Bull.

[28] Sakar O, Sülün T, Kurt H, Gençel B (2011). Reliability and comparison of two facial measurements to detect changes of occlusal vertical dimension in complete denture wearers. Gerodontology.

[29] Raschke GF, Rieger UM, Bader RD, Guentsch A, Schaefer O, Schultze-Mosgau S (2013). Soft tissue outcome after mandibular advancement--an anthropometric evaluation of 171 consecutive patients. Clin Oral Investig.

[30] Raschke GF, Rieger UM, Bader RD, Schultze-Mosgau S (2012). Lip reconstruction: an anthropometric and functional analysis of surgical outcomes. Int J Oral Maxillofac Surg.

[31] Narins RS, Carruthers J, Flynn TC, Geister TL, Görtelmeyer R, Hardas B (2012). Validated assessment scales for the lower face. Dermatol Surg.

